# Psoas tunnel perforation—an unreported complication of hip arthroscopy

**DOI:** 10.1093/jhps/hnv043

**Published:** 2015-06-10

**Authors:** Ryan M. Degen, Eilish O’Sullivan, Ernest L. Sink, Bryan T. Kelly

**Affiliations:** Center for Hip Preservation, Hospital for Special Surgery, 541 East 71st Street, New York, NY 10021, USA

## Abstract

The utilization of hip arthroscopy is rapidly increasing due to improved arthroscopic techniques and training, better recognition of pathology responsible for non-arthritic hip pain and an increasing desire for minimally invasive procedures. With increasing rates of arthroscopy, associated complications are also being recognized. We present a series of six patients who experienced psoas tunnel perforation during anchor insertion from the distal anterolateral portal during labral repair. All patients underwent prior hip arthroscopy and labral repair and presented with persistent symptoms at least partly attributable to magnetic resonance imaging (MRI)-documented psoas tunnel perforation. Their clinical records, operative notes and intra-operative photographs were reviewed. All patients presented with persistent pain, both with an anterior impingement test and resisted hip flexion. MRI imaging demonstrated medial cortical perforation with anchors visualized in the psoas tunnel, adjacent to the iliopsoas muscle. Four patients have undergone revision hip arthroscopy, whereas two have undergone periacetabular osteotomies. All patients had prominent anchors in the psoas tunnel removed at the time of surgery, with varying degrees of concomitant pathology appropriately treated during the revision procedure. Care must be utilized during medial anchor placement to avoid psoas tunnel perforation. Although this complication alone was not the sole cause for revision in each case, it may have contributed to their poor outcome and should be avoided in future cases. This can be accomplished by using a smaller anchor, inserting the anchor from the mid-anterior portal and checking the drill hole with a nitinol wire prior to anchor insertion.

## INTRODUCTION

Traditional hip arthroscopy was initially described using three standard portals—the anterolateral, posterolateral and anterior portals [1, 2]. With increasing experience, surgeons have made slight modifications to these portals to assist with improved visualization and access to both intra- and extra-articular pathology. Currently, the anterolateral and mid-anterior portals are seen as standard by most hip arthroscopists, while many also add a distal anterolateral (DALA) portal to assist with anchor insertion or performing a T-capsulotomy for improved peripheral compartment access. The traditional anterior portal is seldom used, replaced instead by the mid-anterior portal, positioned distal and lateral to the traditional anterior portal, which had been at the point of intersection between a vertical line from the tip of the greater trochanter and a horizontal line running distally from the anterior superior iliac spine (ASIS) [3]. While commonly used for suture anchor insertion during labral repair, this portal uses a trajectory that is convergent to the articular surface with an associated risk of joint perforation when inserting anchors from this portal. As a result, some authors have advocated for the use of percutaneous anchor insertion or insertion from a distal portal, which has been termed the DALA portal [3, 4]. This portal has a more divergent angle from the articular surface to avoid articular penetration, demonstrating excellent utility in anchor placement from 10 o’clock to 2 o’clock positions. However, recently, we have developed concerns over the trajectory from this portal for more medial anchor insertion (i.e. the 3 o’clock position) at the position of the psoas-U, as there is a risk for the anchor to perforate through the anteromedial cortex of the acetabular dome and abut the iliopsoas tendon [5]. This region has previously been referred to as the ‘psoas tunnel’, and represents the area medial to the anterior inferior iliac spine and iliopectineal eminence where the psoas muscle lies [6].

We will report on a series of six patients that presented for assessment and treatment at our hip preservation institution, following prior hip arthroscopy with incomplete symptom resolution. All had experienced medial cortical perforation during anchor insertion from the DALA portal, with anchors perforating into their psoas tunnel following hip arthroscopy and labral repair. To our knowledge, this complication has not yet been reported in the literature. We will outline each patient’s clinical history and operative findings and provide recommendations to avoid this potential complication.

## CASE REPORTS

### Case 1

**Fig. 1.**
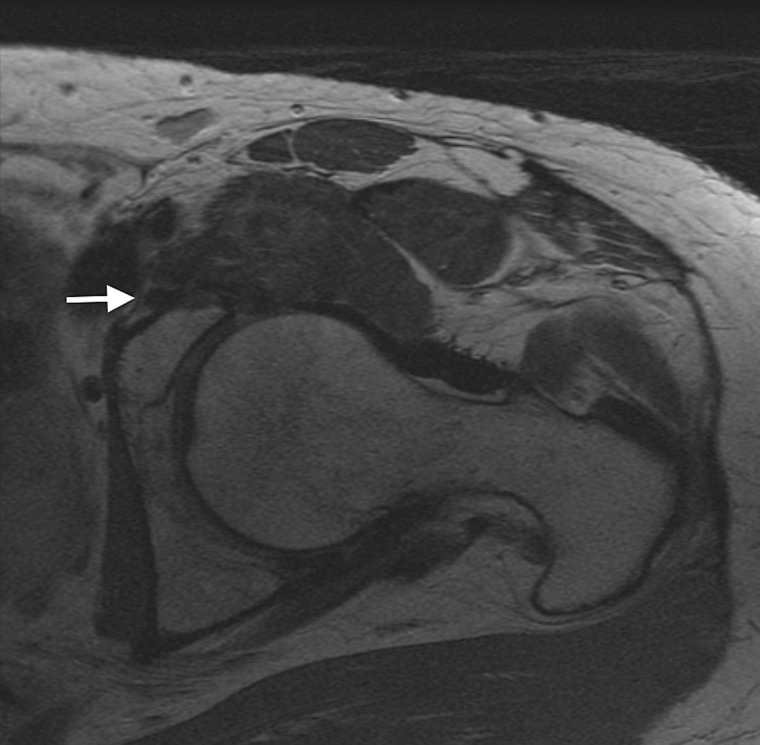
Axial MRI displaying anchor material (white arrow) perforating through the medial cortex, adjacent the neurovascular bundle 68 × 67 mm.

**Fig. 2.**
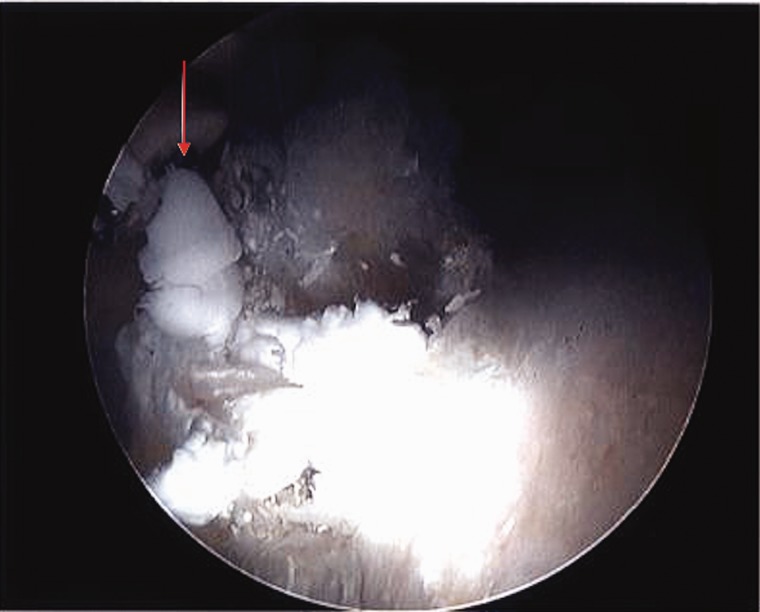
Intra-operative view of psoas tunnel perforation with the tip of the suture anchor breaching the medial cortex 49 × 40 mm.

Case 1 was a 37-year-old female, who presented with 3-year history of left hip pain. She had previously been assessed and treated with a primary hip arthroscopy, undergoing femoral osteoplasty for a cam deformity and repair of an associated labral tear. Following her index procedure, she had incomplete pain relief with hip motion, primarily in terminal hip flexion and underwent a subsequent revision hip arthroscopy 6 months later, although the diagnosis and details of the revision arthroscopic procedure were not available as they were completed at an outside institution. She presented to our clinic 1 year later with residual anterior hip pain. Her clinical exam demonstrated pain provocation with resisted hip flexion, and a positive anterior impingement test. The patient also presented with symptoms of femoral nerve irritation, with complaints of thigh pain and dysesthesia. She underwent further imaging which identified a recurrent labral tear and residual cam impingement. The magnetic resonance imaging (MRI) also identified the presence of anchor material within the psoas tunnel, near the posterior aspect of the neurovascular bundle (Fig. 1). She subsequently underwent a second revision arthroscopic procedure for management of her recurrent labral tear and psoas tunnel perforation. During the revision procedure it was noted that the medial cortex had been perforated during anterior anchor placement, with the anchor tip sitting ∼4.5 mm out of bone and projecting into the psoas tunnel causing inflammation of the iliopsoas (Fig. 2). She underwent removal of this foreign body, selective labral debridement and subspine decompression. She had improvement in pain and function post-operatively, however she continued to have symptoms of femoral nerve irritation at latest follow-up (6 months) (Table I).

**Table I.** Patient demographics and clinical outcome measures

Case	Age	Sex	Duration between primary and revision arthroscopy	Psoas perforation recognized pre-op/intra-op?	Anchor perforation visible on MRI?	Latest follow-up	Clinical outcome measures
1	37	F	15 months	Pre-op	Yes	6 months	Pre-op: HHS 47.3, HOS-ADL 71.88, HOS-SSS 39.29, iHOT-33 34.48
							Post-op: 47.3, HOS-ADL 67.65, HOS-SSS 36.11, iHOT-33 29.87^a^
2	21	M	26 months	Intra-op	No	6 months	Pre-op: HHS 56.1, HOS-ADL 77.94, HOS-SSS 41.67, iHOT-33 46.58
							Post-op: HHS 67.1, HOS-ADL 94.12, HOS-SSS 66.67, iHOT-33 51.96
3	17	M	7 months	Pre-op	Yes	1 years	Pre-op: HHS 59.4, HOS-ADL 75, HOS-SSS 19.44, iHOT-33 29.35
							Post-op: HHS 73.7, HOS-ADL 98.53, HOS-SSS 83.33, iHOT-33 71.26
4	19	F	21 months	Pre-op	Yes	4 months	N/A
5	38	F	N/A	Intra-op	No	4 months	N/A
6	19	F	39 months	Pre-op	Yes	6 months	Pre-op: HHS – 47.3, HOS-ADL 63.24, HOS-SSS 33.33
							Post-op: HHS- 41.8, HOS-ADL 44.12, HOS-SSS 25, iHOT-33 23.7

^a^Complicated by complex regional pain syndrome.

### Case 2

Case 2 was a 21-year-old male collegiate soccer player, who presented with ∼3 years of insidious onset hip pain. He had activity related hip pain, worse with active hip flexion. Two years prior, he was evaluated by another surgeon and underwent a hip arthroscopy with femoral osteoplasty and a labral repair. Although he had temporary improvement, his symptoms gradually worsened as he progressed through rehabilitation. Following rehab, he was limited in his ability to return to sports, with reports of pain and mechanical symptoms (i.e. clicking, locking). Similarly, physical examination revealed pain with resisted hip flexion, and with passive hip extension. He also demonstrated pain with terminal passive flexion consistent with subspine impingement. Repeat imaging revealed a labral tear with residual cam deformity, a prominent subspine and capsular deficiency. During the revision hip arthroscopy, following interportal capsulotomy, loose bodies were encountered. Upon further exploration medially, it was identified that he had loose anchor material within the psoas tunnel, again with medial cortical perforation noted. This material was removed. He subsequently underwent labral repair, cam and subspine debridement with capsular closure. Clinical improvement with symptom resolution was observed during the post-operative period (Table I). It should be noted that the alleviation of his mechanical symptoms was likely attributable to the removal of loose bodies, rather than removal of the prominent anchors in his psoas tunnel.

### Case 3

**Fig. 3.**
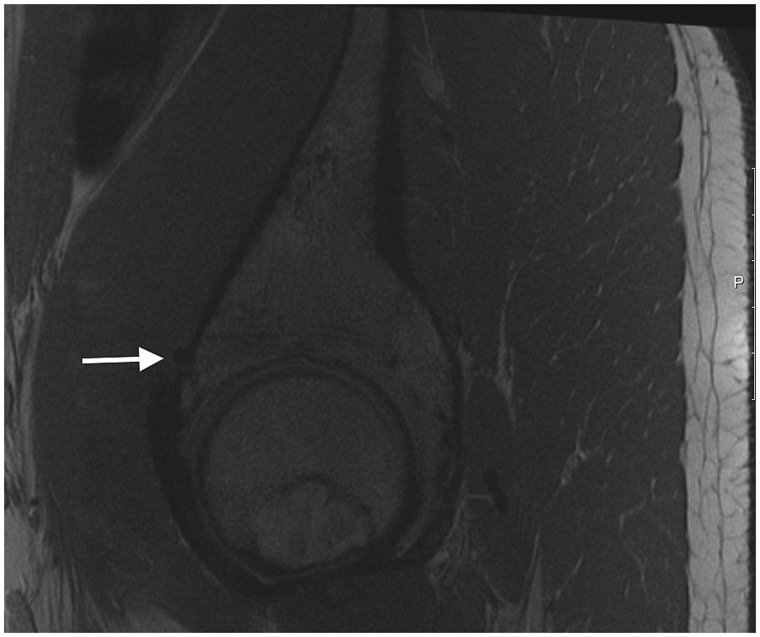
Sagittal MRI demonstrating medial cortical perforation with prominent suture anchor (white arrow) 76 × 64 mm.

**Fig. 4.**
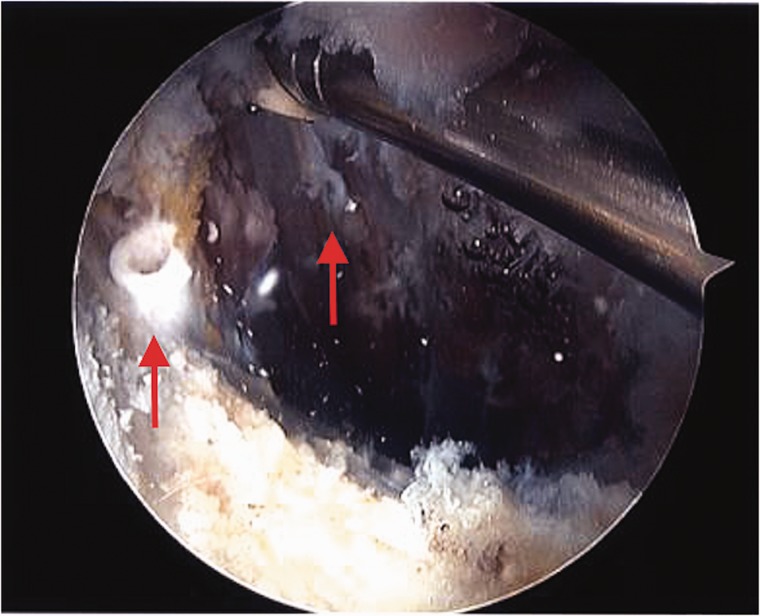
Intra-operative view of free-floating anchors (arrows) within the psoas after displacement following psoas tunnel perforation 49 × 40 mm.

Case 3 was a 17-year-old high school student who ran cross-country. He presented with a 2-year history of groin and hip pain aggravated by sport participation. He was previously assessed by another surgeon and underwent hip arthroscopy for labral repair and bony debridement 1-year prior. His symptoms temporarily improved, however they recurred shortly thereafter, exacerbated by running, kicking and driving, with difficulty returning to sport. Physical examination revealed pain on resisted hip flexion and a positive anterior impingement test. Repeat imaging identified a recurrent labral tear and cam impingement, but also identified anchor perforation through the anteromedial dome, with the anchor impinging on the iliopsoas (Fig. 3). He underwent revision hip arthroscopy, during which the loose anchors were identified abutting the iliopsoas tendon following interportal capsulotomy (Fig. 4). He underwent removal of the anchors, repeat labral repair and femoral osteoplasty with improvement of symptoms and successful return to sport (Table I).

### Case 4

**Fig. 5.**
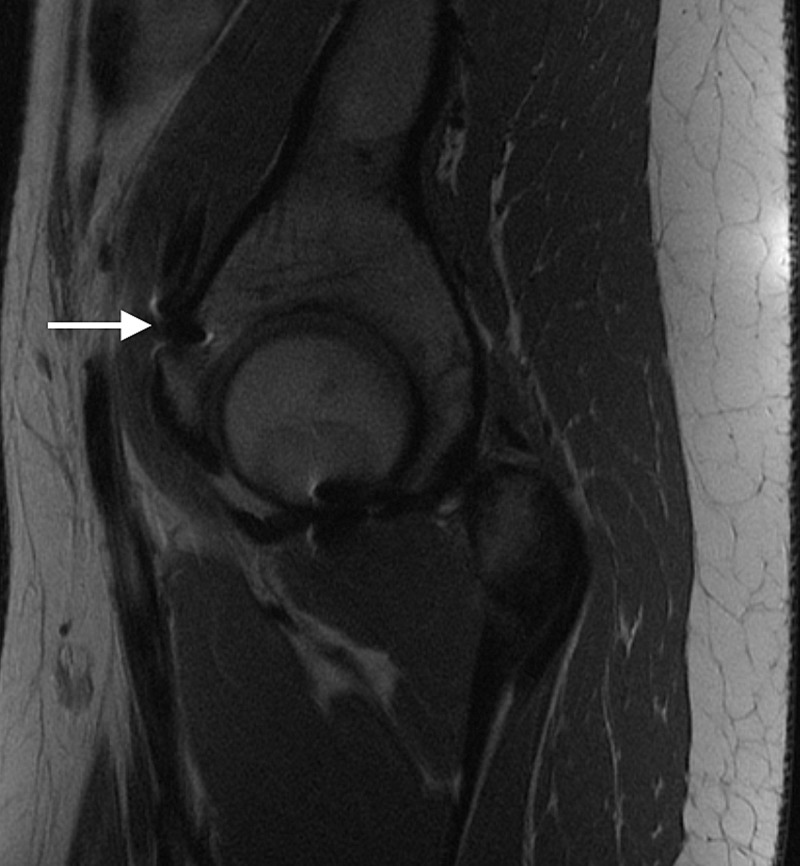
Sagittal MRI demonstrating medial cortical perforation with prominent suture anchor (white arrow) 65 × 70 mm.

**Fig. 6.**
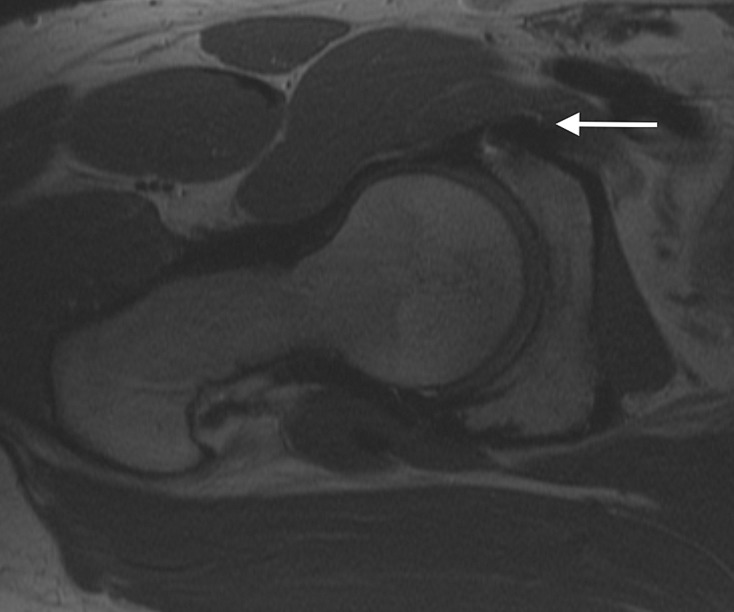
Axial MRI demonstrating medial cortical perforation with prominent suture anchor (white arrow) 77 × 64 mm.

Case 4 is a 19-year-old female who presented with 1 year of hip pain, which was insidious in onset. She had undergone a prior hip arthroscopy with labral repair and bony decompression for femoroacetabular impingement (FAI). Symptoms recurred within 2 months of the procedure and the patient complained of painful snapping in the anterior aspect of the hip. Repeat physical examination confirmed anterior hip pain with resisted hip flexion and passive extension from a flexed position. Imaging identified residual cam impingement, with a prominent anchor causing psoas irritation (Figs. 5 and 6). This patient underwent anchor removal, revision cam debridement and labral refixation with early subjective improvement in pain; however, she is only 4 months post-operatively with no objective outcome data to corroborate this finding.

### Case 5

**Fig. 7.**
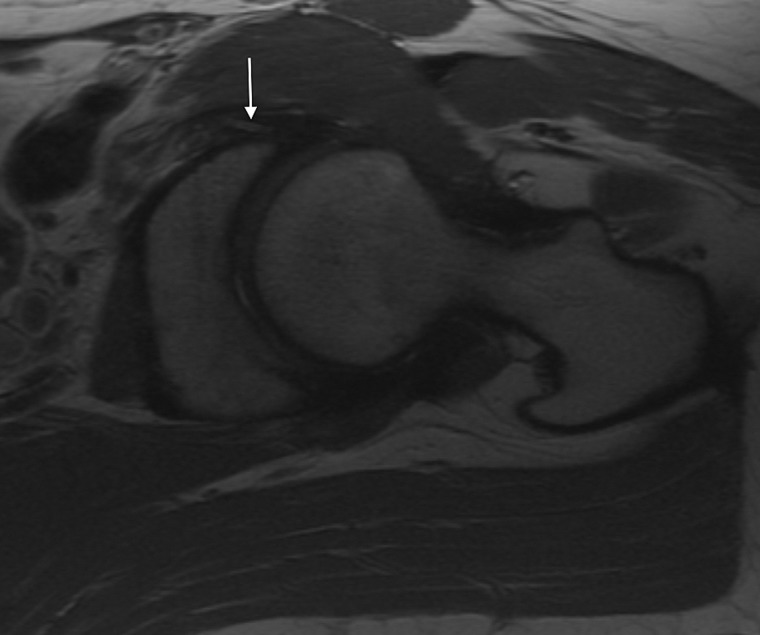
Axial MRI demonstrating medial cortical perforation (arrow) 77 × 64 mm.

**Fig. 8.**
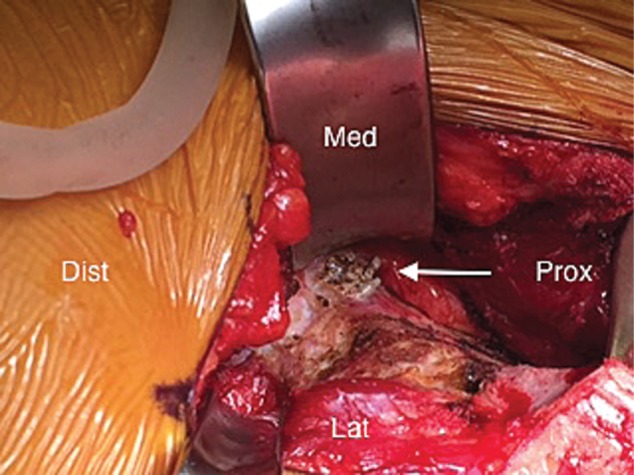
Intra-operative photo during PAO demonstrating prominent medial anchors 27 × 20 mm.

A 38-year-old female presented with a painful left hip. She presented for assessment 5 years prior, where investigations revealed a labral tear. She underwent three subsequent hip arthroscopies, starting with a labral repair, followed by a labral reconstruction and partial psoas release, and finally a capsular plication for her ongoing discomfort, which was felt to relate to micro-instability and capsular deficiency. Unfortunately her symptoms persisted with anterior groin pain, as well as pain with resisted hip flexion. Imaging studies revealed borderline dysplasia with a decreased lateral center-edge angle, a crossover sign and empty posterior wall sign. MRI demonstrated the presence of iliopsoas tendinitis in addition to the dysplastic features, with suture anchor material breaching into the psoas tunnel (Fig. 7). She was subsequently treated with a periacetabular osteotomy to improve her acetabular coverage and reduce her acetabular retroversion, where during the surgical exposure, moderate iliopsoas bursitis was identified and three anchors were found to perforate the medial cortex, projecting nearly 1 cm into the psoas tunnel (Fig. 8). Anchors were removed, and the osteotomy was successfully completed. The patient is ∼4 months out from the surgery, with an uncomplicated early recovery.

### Case 6

Case 6 is a 19-year-old female collegiate soccer player with 4 years of right hip pain. She was initially assessed at an outside facility, and investigations revealed FAI, with a pincer impingement pattern. She underwent previous hip arthroscopy with rim trimming and labral repair with incomplete symptom resolution. She then underwent a second hip scope for repeat osteochondroplasty of the femoral neck and acetabular rim with labral repair. Following this, she presented to our facility with symptoms of residual impingement with a notable cam lesion and underwent a surgical dislocation for osteochondroplasty of her residual impingement. Unfortunately she presented again, several months later with increasing groin pain. MRI and computed tomography scans revealed a recurrent labral tear with acetabular retroversion. On her MRI, we also noticed prominent suture anchor material in the psoas tunnel. She underwent a subsequent combined hip scope and periacetabular osteotomy for repair of her labrum, removal of the prominent anchors and reduction of her acetabular retroversion. She is now 6 months post-operatively with minimal change in clinical outcomes since the procedure (Table I).

## DISCUSSION

While there appears to be improved clinical exposure and training for hip arthroscopy, the increase in utilization of hip arthroscopy in recent years has allowed further study of surgical indications, techniques and complications. Hip arthroscopy has demonstrated that it has a steep learning curve, with several studies identifying decreasing rates of complications with increasing surgeon volume and surgical skill [7–10]. A meta-analysis has even suggested that a minimum of 30 procedures is required to establish competency, although their definition of competency is primarily based on a reduction of procedural complications beyond this volume, and not correlated with improved patient outcomes or technical proficiency [11].

Classically, procedural complications for hip arthroscopy and labral repair could be classified as minor (including iatrogenic chondrolabral injury, traction neuropraxia, superficial infection, instrument breakage, heterotopic ossification) or major (including deep vein thrombosis, deep surgical site infection, pulmonary embolus or intra-abdominal fluid extravasation) [8, 11, 12]. While the rate of these complications is relatively low, reportedly ranging from 1.4% to 7.5% [7, 12, 13], the definition of complications has expanded in recent years. Studies have identified and started reporting on ‘surgical-technique related’ complications, including variables that were previously not considered, such as inadequate or excessive bony resection and equipment failure (i.e. anchor failure). Recently, several studies have identified that inadequate resection, or residual FAI, is a significant complication as it represents the most common reason for revision hip arthroscopy, accounting for up to 75% of the revision arthroscopy cases in some series’ [14, 15].

Although prior literature has accurately quantified the more commonly recognized complications, we have become aware that potentially unrecognized complications of this novel procedure exist by the identification of these surgical-technique related complications. The cases presented in this article represent one such complication and are the first report of psoas tunnel perforation following anchor insertion from the DALA during arthroscopic labral repair. Interestingly, most patients experienced pain with resisted hip flexion, which may be clinically useful in identifying cases of psoas tunnel perforation in patients who have had prior arthroscopic labral repair along the anteromedial rim, at the level of the psoas-U. The symptoms of these patients cannot solely be attributed to this finding as many had concomitant pathology, however psoas tunnel perforation may have contributed to their poor outcome and need for revision surgery, and should be noted as a future consideration and avoided by surgeons during anchor placement. As can be seen by their clinical outcome data (Table I), often their outcomes are unsatisfactory even following revision arthroscopy, and as a result all measures to avoid potential contributing factors to these poor outcomes must be taken, including avoiding this complication. In cases where additional concomitant pathology may be limited, with psoas tunnel perforation as an isolated finding, consideration could be given to utilizing a therapeutic ultrasound-guided iliopsoas bursal injection for management of their hip pain, as success has previously been demonstrated in the setting of a snapping iliopsoas tendon[16, 17].

**Fig. 9.**
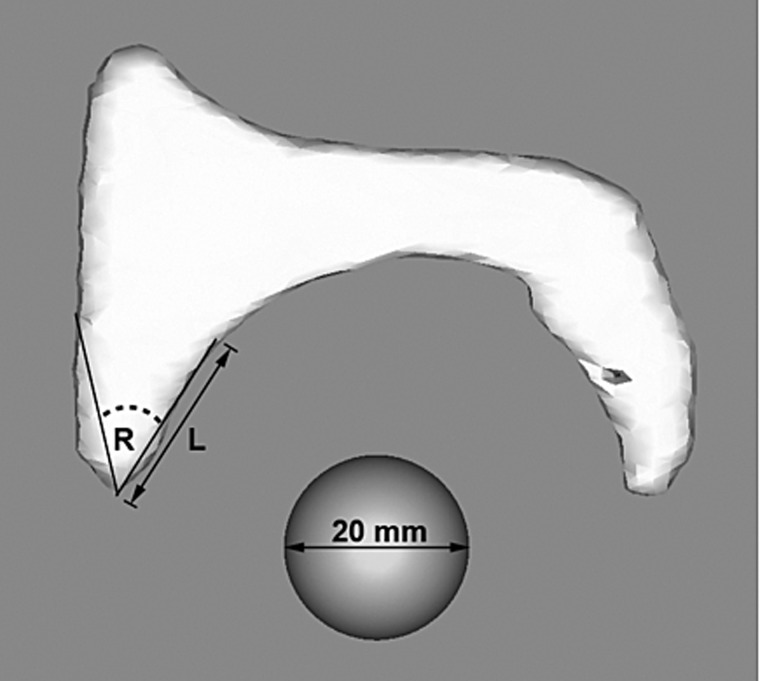
Acetabular rim angle (reprinted with permission, from Ref. 19) 36 × 33 mm.

Some of the challenges with anchor placement during hip arthroscopy, including psoas tunnel and articular perforation, may stem from the aforementioned learning curve before technical proficiency. However the difficulty may also stem from a lack of clinically relevant guidelines for optimal anchor insertion angles and positions relative to the acetabular rim. Philippon *et al*. [18] proposed an insertion angle of 15° relative to the vertical when inserting anchors from the anterolateral portal, however this is specific to patient position (supine on fracture table), with no reference point of proximity to the acetabular edge or labral edge provided, limiting the generalization of this recommendation. Lertwanich *et al*. [19] attempted to define a safety margin for anchor insertion following acetabular rim trimming using the acetabular rim angle. This angle is the arc defined by two lines drawn from the same start point, 2–3 mm adjacent to the chondrolabral junction, with one extending to the subchondral bone at the articular surface and the other to the outer cortex of the acetabulum, with each line 20 mm in length to simulate the drill length for anchor insertion (Fig. 9). This effectively provides the degree of variability in the angle of insertion that allows the anchor to remain safely in bone. They identified that this angle was smallest at the anterosuperior quadrant, or 3 o’clock position, consistent with the location where we noted psoas tunnel perforation, and recommended caution be utilized in anchor placement in this position as the acetabular safety angle is the smallest here [19]. They also noted that the depth of the drill played a role, with longer drill bits having a narrower safety margin, or smaller acetabular angle. Trimming the acetabular rim was also shown to help increase the acetabular angle.

Hernandez and McGrath [20] also tried to clarify the safe-angle for anchor insertion by comparing the trajectory of anchor insertion relative to a perpendicular to the acetabular face. They also attempted to quantify the position that the anchor should be started relative to the rim of the acetabulum. In their recommendations, they identified a target angle of 10° relative to a perpendicular to the acetabular face, having the anchor slightly convergent to the acetabulum to avoid outer cortex perforation. They also suggested a start point 2.3–2.6 mm from the acetabular rim for insertion of anchors that are 3.0 mm or smaller [20]. They point out that smaller drill bits, and therefore smaller anchors, had a greater safe angle of insertion, as noted in Lertwanich’s study as well [20].

In addition to the aforementioned findings and recommendations to reduce the risk of either psoas tunnel perforation or articular perforation, we recommend an additional step of using the nitinol wire, commonly used for portal establishment, to check all potentially high-risk drill holes to ensure that they are contained and have not perforated into the psoas tunnel or joint after drilling and before anchor insertion. In addition, consideration could be given to switching to the mid-anterior portal for anchor insertion at the higher-risk 3 o’clock position, as this may represent a more favorable trajectory avoiding medial cortical perforation at this location.

While the above refer to technical components of the procedure that contribute to complications of anchor insertion, we recognize that additional variables, including surgeon bias, available equipment and patient anatomy, may also contribute to the rates of ‘surgical-technique’ related complications. As we have highlighted earlier, portal use is variable, with some surgeons preferring to place anchors from the mid-anterior portal, others using the DALA portal, and others using a combination of the two, which can significantly alter drill trajectory to potentially alter rates of joint or psoas tunnel perforation. In addition to variability in portal use, the use of either a straight or curved drill guide can also affect complication rates during anchor insertion, as demonstrated by Nho *et al.* [21], where they found that the use of a curved drill guide from the DALA portal improved the safety of anchor insertion at the 1 o’clock position compared with a straight drill guide [21]. Finally, patient anatomy, including femoral and acetabular version as well as acetabular depth, may also influence portal placement and anchor insertion along the acetabular rim, contributing to the complication rates associated with this step. With all of these different factors contributing to complications during anchor insertion, the recommendations presented in this discussion cannot be followed in isolation, but must instead be considered in the context of one another to limit the rate of complications.

This report represents the first to identify and raise awareness of this heretofore-unreported complication. While we recognize these cases represent a select few from greater than 3500 hip arthroscopy cases, we feel that surgeons should be aware of this potential complication in order to avoid similar cases, although we recognize that psoas anchor perforation alone cannot be considered the causal factor for failure of the index procedure in these cases as there was a high rate of concomitant pathology. However, this case series highlights the need for further study of the techniques utilized for anchor insertion to determine optimal methods to limit potential complications going forward.

## CONCLUSIONS

Caution should be exercised when inserting anchors in the anterosuperior quadrant as the safe-angle for insertion is narrowest at this position, and there is an increased risk for psoas tunnel perforation when inserting anchors from the DALA with a straight drill guide. Using smaller anchors with shorter drill lengths, checking drill holes for evidence of perforation with a nitinol wire, and consideration for anchor insertion from alternate portals at this position, could all potentially help to reduce rates of psoas tunnel perforation.

## CONFLICT OF INTEREST STATEMENT

None declared.
